# The multimodal Munich Clinical Deep Phenotyping study to bridge the translational gap in severe mental illness treatment research

**DOI:** 10.3389/fpsyt.2023.1179811

**Published:** 2023-05-05

**Authors:** Lenka Krčmář, Iris Jäger, Emanuel Boudriot, Katharina Hanken, Vanessa Gabriel, Julian Melcher, Nicole Klimas, Fanny Dengl, Susanne Schmoelz, Pauline Pingen, Mattia Campana, Joanna Moussiopoulou, Vladislav Yakimov, Georgios Ioannou, Sven Wichert, Silvia DeJonge, Peter Zill, Boris Papazov, Valéria de Almeida, Sabrina Galinski, Nadja Gabellini, Genc Hasanaj, Matin Mortazavi, Temmuz Karali, Alexandra Hisch, Marcel S Kallweit, Verena J. Meisinger, Lisa Löhrs, Karin Neumeier, Stephanie Behrens, Susanne Karch, Benedikt Schworm, Christoph Kern, Siegfried Priglinger, Berend Malchow, Johann Steiner, Alkomiet Hasan, Frank Padberg, Oliver Pogarell, Peter Falkai, Andrea Schmitt, Elias Wagner, Daniel Keeser, Florian J. Raabe

**Affiliations:** ^1^Department of Psychiatry and Psychotherapy, University Hospital, LMU Munich, Munich, Germany; ^2^International Max Planck Research School for Translational Psychiatry (IMPRS-TP), Munich, Germany; ^3^Department of Ophthalmology, University Hospital, LMU Munich, Munich, Germany; ^4^Department of Psychiatry and Psychotherapy, University Medical Center Göttingen, Göttingen, Germany; ^5^Department of Psychiatry and Psychotherapy, Otto-von-Guericke-University Magdeburg, Magdeburg, Germany; ^6^Laboratory of Translational Psychiatry, Otto-von-Guericke-University Magdeburg, Magdeburg, Germany; ^7^Center for Behavioral Brain Sciences, Magdeburg, Germany; ^8^Center for Health and Medical Prevention, Magdeburg, Germany; ^9^Department of Psychiatry, Psychotherapy and Psychosomatics of the University Augsburg, Medical Faculty, University of Augsburg, Augsburg, Germany; ^10^Max Planck Institute of Psychiatry, Munich, Germany; ^11^Laboratory of Neurosciences (LIM-27), Institute of Psychiatry, University of São Paulo, São Paulo, Brazil; ^12^NeuroImaging Core Unit Munich, University Hospital, LMU Munich, Munich, Germany; ^13^Munich Center for Neurosciences, LMU Munich, Munich, Germany

**Keywords:** schizophrenia, research domain criteria, retina, electrophysiology, multimodal magnetic resonance imaging, electroencephalography

## Abstract

**Introduction:**

Treatment of severe mental illness (SMI) symptoms, especially negative symptoms and cognitive dysfunction in schizophrenia, remains a major unmet need. There is good evidence that SMIs have a strong genetic background and are characterized by multiple biological alterations, including disturbed brain circuits and connectivity, dysregulated neuronal excitation-inhibition, disturbed dopaminergic and glutamatergic pathways, and partially dysregulated inflammatory processes. The ways in which the dysregulated signaling pathways are interconnected remains largely unknown, in part because well-characterized clinical studies on comprehensive biomaterial are lacking. Furthermore, the development of drugs to treat SMIs such as schizophrenia is limited by the use of operationalized symptom-based clusters for diagnosis.

**Methods:**

In line with the Research Domain Criteria initiative, the Clinical Deep Phenotyping (CDP) study is using a multimodal approach to reveal the neurobiological underpinnings of clinically relevant schizophrenia subgroups by performing broad transdiagnostic clinical characterization with standardized neurocognitive assessments, multimodal neuroimaging, electrophysiological assessments, retinal investigations, and omics-based analyzes of blood and cerebrospinal fluid. Moreover, to bridge the translational gap in biological psychiatry the study includes *in vitro* investigations on human-induced pluripotent stem cells, which are available from a subset of participants.

**Results:**

Here, we report on the feasibility of this multimodal approach, which has been successfully initiated in the first participants in the CDP cohort; to date, the cohort comprises over 194 individuals with SMI and 187 age and gender matched healthy controls. In addition, we describe the applied research modalities and study objectives.

**Discussion:**

The identification of cross-diagnostic and diagnosis-specific biotype-informed subgroups of patients and the translational dissection of those subgroups may help to pave the way toward precision medicine with artificial intelligence-supported tailored interventions and treatment. This aim is particularly important in psychiatry, a field where innovation is urgently needed because specific symptom domains, such as negative symptoms and cognitive dysfunction, and treatment-resistant symptoms in general are still difficult to treat.

## 1. Introduction

Over the last century, advances in psychopharmacological medication have improved the outcome of severe mental illnesses (SMIs), including schizophrenia (SZ), bipolar disorder (BD), and major depressive disorder (MDD) (1). However, despite these efforts, SMIs remain debilitating and have a high global disease burden because they first manifest usually in young adults and a third to a half of patients continue to experience symptoms even after they fulfill criteria for remission (2–5). Moreover, response to pharmacological interventions is highly variable (6), and a substantial number of individuals develop treatment resistance early in the course of an SMI (7). Treatment resistance is defined as reduced or non-response to an adequate treatment and is associated with increased healthcare burden, although in some disorders, criteria for treatment resistance still vary (8). A recent meta-analysis found rates of almost 25% for early treatment resistance in first-episode psychosis and SZ (7). Treatment-resistant schizophrenia (TRS) is defined as “nonresponse to at least 2 sequential antipsychotic trials of sufficient dose, duration, and adherence” (9). A 5-year prospective evaluation of outcome in individuals with a first-episode of a schizophrenia spectrum disorder (SSD, *N* = 246) found that 23% were treatment resistant from the start of the illness and that this was also the case in 70% of those with treatment resistance (10). Two types of TRS have been defined (10, 11): primary TRS, i.e., SZ that shows treatment resistance from the start of antipsychotic treatment, and secondary TRS, i.e., SZ where antipsychotics have initial effects but patients later develop TRS (9).

In scientific and clinical communities, the most widely accepted definition of treatment-resistant depression is a depressive episode that shows “a minimum of two prior treatment failures and confirmation of prior adequate dose and duration” (12). Defining treatment resistance in BD is challenging because course episodes are not uniform but have a complex clinical picture and complex treatment options (13). Some patients do not tolerate therapeutic trials or are noncompliant and are referred to as “pseudorefractory” (13). Treatment-resistant BD is defined as a “failure of symptoms improvement despite an adequate trial of two therapeutic agents” (14). Better knowledge about the neurobiological background of treatment resistance is urgently needed.

Before the revolutionary advances of molecular genetics, epidemiological studies already observed that first-degree relatives of SZ patients had a 10% lifetime risk to develop SZ, in contrast to the 1% risk in the general population. Therefore, the best-known risk factor for SZ is first-degree positive family history (15). The genetic heritability is estimated to be about 79% for SZ and 73% for SSD (16), and over the last decade, genome-wide association studies (GWASs) have found over 270 risk loci for SZ (17). The liability-based single nucleotide polymorphism (SNP) heritability (SNP-h^2^, i.e., additive genetic variance explained by all SNPs) has been estimated to be 18.6% for BD and 24% for SZ (17, 18). A recent meta-analysis of GWASs in various mental disorders showed a strong genetic correlation (*r*_g_) between SZ and BD (*r*_g_ = 0.70) and SZ and MDD (*r*_g_ = 0.34) (19). In addition, it showed that of the formerly 109 pleiotropic genome-wide loci identified in psychiatric traits, 83% were associated with SZ, 72% with BD, and 48% with MDD. Moreover, environmental factors, such as complications during childbirth, trauma during childhood, urban living, migration, and abuse of cannabis (20), are suggested to be part of the dynamic interplay that leads to the onset of SMI on the basis of a high-risk genetic background (21).

Despite the above, the pathophysiological background of SMIs is only poorly understood. The age of onset in SZ and BD is mostly during adolescence and early adulthood, i.e., during phases in which neurodevelopment switches from the production of new synapses to synaptic pruning, in which the number of synapses is reduced. In SMIs, neurodevelopmental disturbances may lead to synaptic deficits in connected brain regions (21–25), which could partly explain the deficits in connectivity and gray matter loss seen in SMIs (26, 27).

Besides the enlargement of ventricles, studies on SMI have also detected volume loss in corticostriatal-thalamic networks, which include the dorsolateral prefrontal cortex and temporal, parietal, and limbic regions (28, 29). A meta-analysis of more than 16,000 patients across psychiatric disorders and healthy controls (HC) showed alterations of gray matter volumes and resting-state functional connectivity in salience network areas, such as the anterior cingulate cortex and left and right insula, and in the default mode network, including the anterior cingulate cortex and frontoparietal cortex, providing evidence for a biologically driven transdiagnostic marker in SMIs (30, 31). Mature neuronal circuits are essential for brain functioning and required for higher cognitive processes such as attention and working memory, which are maintained by synchronized neuronal oscillations, especially at approximately 40 Hertz (gamma oscillations) (32). In SMIs, dysfunctional gamma oscillations are the basis of cognitive dysfunction and may be associated with an excitatory/inhibitory imbalance (33–35). However, despite the neurobiological background of SMIs, their diagnosis according to the Diagnostic and Statistical Manual of Mental Disorders, 5th Edition (DSM-5), and the International Classification of Diseases, 10th revision (ICD-10), is still based only on operationalized, symptom-based clusters, and there is a great need to identify biomarkers for each individual SMI (36).

SMIs are considered to be highly heterogeneous. For example, impairments in cognition are often thought to be present in all SMIs, but in clinical practice, only a subgroup of patients is affected by severe cognitive impairments and other subgroups have good cognitive performance (37). However, the underlying differences in neurobiology between those subgroups remain unclear. Another topic of discussion is whether pathophysiologic processes are the same in treatment-responsive and treatment-resistant individuals or whether these processes are more severe or progressive in treatment-resistant patients and whether treatment resistance is, at least partially, pathophysiologically distinct or even a transdiagnostic phenomenon (8). Noteworthy in this context is that psychiatric diagnoses often shift over time (38). Moreover, some individuals reach a state of remission relatively soon after an exacerbation of first-episode psychosis, whereas others report persisting symptoms (39).

Affective disorders such as MDD and BD not only clinically overlap with SSDs (38) but are also on a polygenetic spectrum (40–42). This commonality may explain why existing drug treatments for SMIs like MDD or SZ are often beneficial in a broader spectrum of diseases and supports the theory that transdiagnostic phenomenological approaches might help to reveal the underling neurobiology (43).

Hence, DSM-5 and ICD-11 do not reflect findings from the fields of genetics and neuroscience, but these findings should be considered when developing treatment approaches in terms of biology-based, individualized precision medicine (43). In 2010, the US National Institute of Mental Health launched the Research Domain Criteria (RDoC) (44), a neurobiology-based, research-orientated classification framework that investigates mental health and pathological states of six neurobehavioral major *domains* (negative valence systems, positive valence systems, cognitive systems, social processes, arousal/regulatory systems, and sensorimotor systems) and their *(sub)constructs* (e.g., attention, perception, declarative memory, language, cognitive control, and working memory for the cognitive domain) within a full functional range of variation from abnormal to normal^1^ by using various clinical and translational neuroscience tools that are termed *units of analysis*.^2^ The RDoC *units of analysis* include, e.g., genetic analyzes, electrophysiology, multimodal imaging, and neurocognitive assessments. The RDoC reflect mental disorders from a bottom-up, translational perspective (from genes to behavior) and use a transdiagnostic approach (43). This method is in contrast to the DSM-5 and ICD-10 top-down approach, which differentiates between “healthy” states and various “pathological” ones (43). By systematically assessing RDoC *domains* with neuroscience tools, the RDoC initiative aims to improve the diagnostic approach by identifying biotype-informed (sub)groups that may pave the way toward subgroup-specific treatment in psychiatry (43).

Ongoing discussions are considering whether and how RDoC-based research could fit into a clinical environment that uses DSM-5 and ICD-11, whether the use of RDoC would limit translational communication, and whether a more psychopathology-based transdiagnostic classification system such as the Hierarchical Taxonomy of Psychopathology (45) would be even more beneficial (46). However, the RDoC system does not aim to replace the existing clinical classification systems, and combining RDoC with established clinical classification systems might enable (RDoC-based) neurobiological dissection with clinically meaningful outcomes and thus be beneficial for both affected individuals with ongoing symptoms despite adequate treatment and clinicians confronted with fluid diagnoses over time and heterogeneous symptoms despite identical disease entities (47). This approach may also help to develop biomarker-based stratification strategies for identifying clinically meaningful subgroups of patients and thus pave the way for personalized and tailored neurobiologically informed clinical trials and interventions (48–50).

Therefore, the multimodal Clinical Deep Phenotyping (CDP) study at the Department of Psychiatry, University Hospital, LMU Munich, Munich, Germany, aims to apply the RDoC framework in a broad naturalistic and transdiagnostic approach in a cohort of patients with MDD, BD, SSD, and HC, to gain a deeper understanding of the underlying neurobiology of SMI. To do so, it will investigate the existing disease hypotheses (disturbed circuits, brain volume loss, impaired connectivity, dysregulated excitation-inhibition ratio, inflammation, and neuroinflammation) of SMI and address the question whether certain clinically relevant subpopulations (e.g., those with certain clinical outcomes, such as cognitive impairment, those who fulfill Positive and Negative Syndrome Scale [PANSS] remission criteria (51) or have treatment resistance, or those with patient-reported outcomes, such as real-life functioning) are represented in neurobiological biotypes defined with available clinical and translational neuroscience methods. To enable the identification of clinically relevant subgroups, we aim to perform deep phenotyping in over 500 participants with SMI and in over 500 HC. Here, we report on the protocol of the multimodal CDP study and also show the feasibility of the applied multimodal characterization by presenting results in 381 participants who were enrolled in the initiation phase, October 1, 2020, to October 31, 2022.

## 2. Materials and methods

After being approved by the local ethics committee at the LMU Munich, Germany (project number 20–528), the CDP study was initiated as a naturalistic, prospective, single-center study at the Department of Psychiatry and Psychotherapy, University Hospital, LMU Munich, Munich, Germany. The study is registered in the German Clinical Trials Register (ID: DRKS00024177). Data handling in the CDP study is embedded into the Munich Mental Health Biobank (52) of the LMU Munich (project number: 18–716) and uses their approved data storage and data safety concept. The CDP study includes multilayer, transdiagnostic assessments (Table 1; Supplementary Table S1), which are described in more detail below. To enable a transdiagnostic approach, all assessments are performed in all study participants, including HC.

**Table 1:** Evaluation plan.

Evaluations	
Clinical characterization	*
Psychiatric history	*
Physical examination	*
Transdiagnostic self-ratings	CTQ-Screen, Brief Resilience Scale, Loneliness Scale, Lubben Social Network Scale, WHO-5, PHQ-9, MCTQ, WHOQOL-BREF, GAF, CGI
Disease-related scales	PANSS, PANSS RSWG criteria, CDSS, YMRS, IDS-C30
Cognitive assessment	BACS
Cerebral assessment	MRI, EEG, TMS
Retinal assessment	OCT, OCT-A, ERG
Biobanking (Munich Mental Health Biobank)	*

BACS, Brief Assessment of Cognition in Schizophrenia; CDSS, Calgary Depression Rating Scale for Schizophrenia; CGI, Clinical Global Impression; CTQ-Screen, Childhood Trauma Screener; EEG, electroencephalography; ERG, electroretinogram; GAF, Global Assessment of Functioning; IDS-C30, Inventory of Depressive Symptomatology-clinician-rated version with 30 items; MCTQ, Munich Chronotype Questionnaire; OCT, optical coherence tomography; OCT-A, optical coherence tomography angiography; PANSS, Positive and Negative Syndrome Scale; PHQ-9, Patient Health Questionnaire-9; RSWG, Remission in Schizophrenia Working Group; TMS, transcranial magnetic stimulation; WHO-5, Well-Being Index scale; WHOQOL-BREF, WHO-Quality of Life Scale; YMRS, Young Mania Rating Scale¸ MRI, multimodal magnetic resonance imaging.

* See Supplementary Table S1 for details.

### 2.1. Study recruitment and inclusion and exclusion criteria

The cross-diagnostic CDP study includes patients with a diagnosis of SSD, e.g., SZ, schizoaffective disorder (SZA), brief psychotic disorder (BrPsyD), drug-induced psychosis (DIP), and delusional disorder (DD); patients with a diagnosis of BD and MDD; and individuals without a past or current psychiatric disorder (HC). Patients are diagnosed with the Mini-International Neuropsychiatric Interview (M.I.N.I.) (53) according to the DSM-5, text revision (DSM-5-TR, Version 7.0.2), and ICD-10. All participants are aged 18 to 65 years and fluent in German [German language skills are required for the cognitive assessment with the Brief Assessment of Cognition in Schizophrenia (BACS, German version)] (54, 55).

Patients with a primary psychiatric disorder other than SSD, BD, and MDD, candidates younger than 18 years or older than 65 years, pregnant women, and patients with a concurrent clinically relevant neurological or neuropsychiatric disorder that affects the central nervous system (CNS; e.g., epilepsy, stroke, multiple sclerosis, dementia, meningitis, encephalitis, structural brain deficits, and organic psychosis/mania) or other severe somatic comorbidities are excluded. Additional exclusion criteria are the inability to provide written informed consent and relevant non-compliance that would interfere with the ability to participate in the study.

Participants are screened for inclusion and exclusion criteria, and written informed consent is obtained before any study-related procedures are performed.

### 2.2. Clinical assessments

The CDP assessments include the basic Munich Mental Health Biobank phenotyping, which comprises (1) a structured assessment that records socioeconomic background and psychiatric and medical history and screens for a family history of psychiatric disorders and (2) the following transdiagnostic self-ratings: Childhood Trauma Screener (CTQ-Screen) (56), Brief Resilience Scale (57), Loneliness Scale (58), Lubben Social Network Scale (59), World Health Organization-5 Well-Being Index (WHO-5) (60), World Health Organization Quality of Life Scale, abbreviated version (WHOQOL-BREF) (61), Patient Health Questionnaire - 9 (PHQ-9) (62), and Munich Chronotype Questionnaire (MCTQ) (63).

The specific CDP phenotyping includes an additional battery of structured assessments, ratings, examinations, and self-ratings that are performed or administered by trained mental health professionals. If available and applicable, electronic medical records are used to verify the collected data. Medical history includes age at first symptom onset, age at first psychotic, depressive, or manic episode, duration of illness, duration of untreated illness, time of first contact with the mental health care system, number and duration of illness episodes, number of past hospitalizations because of mental illness, and information on whether the current episode is the first one. The phenotyping also includes a structured assessment of current and lifetime psychiatric medication, including previous or current treatment with clozapine (the first-line medication for treatment-resistant SZ) (64) or ketamine; dosages of current antipsychotic medications are transformed into chlorpromazine equivalents (65). In addition, previous or current electroconvulsive therapy is assessed.

The clinical assessment covers the assessment of any past and current physical comorbidities, including CNS conditions, cardiometabolic conditions, and risk factors (i.e., body mass index, resting heart rate, blood pressure, and smoking status), and ophthalmological conditions that may potentially affect vision. Moreover, medication prescribed for physical illnesses is recorded. Cardiovascular risk scores, such as the Prospective Cardiovascular Münster (PROCAM) Score (66) and the body mass index-based Framingham Risk Prediction Score (FRPS) (67), are calculated. The intensity of physical addiction to nicotine is assessed with the Fagerström test (68). Handedness is assessed with the short form of the Edinburgh Handedness Inventory (69), and any shift work or time zone crossings with a time difference of more than 2 h within the last month is noted.

### 2.3. Psychometrics

To enable a transdiagnostic approach, all study participants (including HC) undergo a battery of psychometric tools, independent of the DSM-5-TR and ICD-10 psychiatric diagnosis. Thus, SZ symptoms are assessed in all participants by the PANSS (70). Remission is evaluated on the basis of the PANSS Remission in Schizophrenia Working Group (RSWG) items without the time criterion (“Andreasen criteria”) (51). The Calgary Depression Rating Scale for Schizophrenia (CDSS) (71), the Inventory of Depressive Symptomatology version with 30 items (IDS-C30), clinician-rated version (72), and the Young Mania Rating Scale (YMRS) (73) are also used to assess affective symptoms in all participants.

Global disease severity is evaluated with the Clinical Global Impression (CGI) scale (74), and level of general functioning, with the Global Assessment of Functioning (GAF) scale (74).

### 2.4. Neurocognitive assessment

To assess study participants’ neurocognitive performance within a feasible time (about 30–45 min), we use the BACS battery (55), which covers multiple cognitive domains that are characteristically impaired in psychosis, such as verbal memory, working memory, motor speed, attention, executive functions, and verbal fluency.

### 2.5. Multimodal brain imaging

Multimodal magnetic resonance imaging (mMRI) is performed with a Siemens Magnetom Prisma 3 T MRI scanner (Siemens Healthineers, Erlangen, Germany) and includes anatomical MRI measurements, i.e., T1-weighted magnetization prepared-rapid acquisition gradient echo (T1-MPRAGE), T2 sampling perfection with application-optimized contrasts using different flip angle evolution (T2-SPACE), T2-weighted-fluid-attenuated inversion recovery (T2-FLAIR), and diffusion tensor imaging (DTI), and functional MRI measurements, i.e., resting-state functional MRI (rsfMRI), task-based functional MRI (fMRI), and magnetic resonance spectroscopy (MRS) (Supplementary Figure S1). The Human Connectome Project (HCP) protocol (75) is used for the mMRI measurements; detailed imaging parameters can be found in Supplementary Table S2. In addition, single-voxel spectroscopy is used to collect data at the left dorsolateral prefrontal cortex (DLPFC)/insula and anterior cingulate cortex (ACC). Task-based fMRI uses an HCP visuomotor task; we chose this task to allow comparability of task-based fMRI with other CDP modalities, such as the eye examinations and motor evoked potentials (MEPs) assessed by transcranial magnetic stimulation (TMS, see also section 2.7).

### 2.6. Electroencephalography

Study participants undergo digitized electroencephalography (EEG) recordings lasting approximately 30 min. Recordings are performed with a standardized set-up (BrainAmp amplifier, Brain Products, Martinsried, Germany) with 32 scalp electrodes (10/20 system). After resting-state EEG has been recorded with eyes closed for 5 min and open for 5 min, activation EEG is recorded with an auditory stimulus (P300) (76, 77) for an additional 18 min.

### 2.7. Transcranial magnetic stimulation

For the diagnostic TMS, participants are examined in a half-reclined seated position. For surface electromyography (EMG), electrodes are placed on the first dorsal interosseous muscle of the right hand. Raw EMG signals are amplified and bandpass filtered (2 Hz-3 kHz) with a Digitimer D-360 amplifier (Digitimer Ltd., Welwyn Garden City, United Kingdom), digitized at 5 kHz, and then processed with Signal Software (version 5, Cambridge Electronic Design, Cambridge, United Kingdom). TMS-induced MEPs are evoked by stimulating the left primary motor cortex (M1) with a flat figure-eight coil (outer diameter: 70 mm) connected to a Magstim Bistim2 stimulator (Magstim Company Ltd., Whitland, United Kingdom). Different cortical excitability parameters are investigated with different TMS protocols that use single and paired pulses. More specifically, resting motor threshold, the intensity required to evoke a 1 mV MEP, short-and long-interval intracortical inhibition, and intracortical facilitation are assessed in each participant (Supplementary Table S3). TMS is performed according to established international safety guidelines (78), and each participant undergoes a screening TMS questionnaire prior to participating (79).

A smaller sample of patients with SZ or MDD and some HC undergo simultaneous TMS-fMRI examination. In a test–retest design, the left DLPFC is stimulated with a 10-Hz repetitive TMS protocol with intensities of 40 and 80% of the resting motor threshold. Simultaneous TMS-fMRI is a new technique that enables more causal interpretations of the blood oxygenation level-dependent response (80).

### 2.8. Retinal anatomy and electrophysiology

From a developmental perspective, the retina is part of the brain and therefore considered as an accessible “window to the brain” (81). Moreover, pioneer studies and meta-analyzes have reported retinal alterations in psychiatric disorders (82–85). Therefore, CDP phenotyping includes an assessment of retinal anatomy by optical coherence tomography (OCT), of retinal microvasculature by OCT angiography (OCT-A), and of retinal electrophysiology by electroretinography (ERG). Before the retinal assessments, refraction and visual acuity are determined with an OCULUS/NIDEK AR 1-s autorefractor (OCULUS Optikgeräte GmbH, Wetzlar, Germany) and intraocular pressure is measured with an OCULUS/NIDEK Tonoref II (OCULUS Optikgeräte GmbH, Wetzlar, Germany). OCT and OCT-A are performed on a ZEISS CIRRUS HD-OCT 5000 with AngioPlex (Carl Zeiss Meditec AG, Jena, Germany), and ERG is performed with a mobile RETeval electroretinograph (LKC Technologies, Inc., Gaithersburg, MD, United States).

### 2.9. Overlap with previous deep phenotyping and translational studies

To enable longitudinal and translational investigations to be performed right at the start of the CDP study, we invited those participants from previous deep phenotyping studies at the Department of Psychiatry and Psychotherapy, LMU Munich, who had agreed to be re-contacted for new studies at the Department to participate in the CDP study. These individuals had participated in one or both of the following studies: (a) the Multimodal Imaging in Chronic Schizophrenia Study (MIMICSS), a pilot study that was part of the longitudinal PsyCourse study (86, 87) (local ethics committee of the LMU Munich, Munich, Germany, project no. 17–13; see Supplemental Text), and (b) an add-on study of PsyCourse that established a cohort of donors of human induced pluripotent stem cells (hiPSCs) [ethics committee project no. 17–880; (88)].

### 2.10. Biobanking in the CDP study

The Munich Mental Health Biobank (52) provides the biobanking of samples in the CDP study. For all participants, blood-based biobanking comprises the following: 1 × 7.5 ml K3EDTA Monovette (Fa Sarstedt, Cat no 01.1605.001) for DNA extraction, 1 x PaxGene blood RNA tube (Fa BD, Cat no 762165) for RNA extraction, 1 × 9 ml K3EDTA Monovette (Fa Sarstedt, Cat no 02.1066.001) for plasma-based analysis, and 1 × 9 ml Monovette with coagulation activator (Fa Sarstedt, Cat no 02.1063.001) for serum-based analysis; after initial processing, all samples are stored at −80°C. If laboratory capacities allow additional biobanking, additional vials (BD Vacutainer 10 ml Glass Sodium Heparin Tubes, BD, Cat no 368480) are used for isolating peripheral blood mononuclear cells (PBMC) and stored in liquid nitrogen; the banking of PBMCs in liquid nitrogen enables later generation of hiPSCs (89). We also collect cerebrospinal fluid (CSF) from patients with psychosis in whom a diagnostic lumbar puncture is clinically recommended.

### 2.11. Genetic and epigenetic analyzes

To assess the genetic risk background of these individuals, the DNA isolated during biobanking of the samples will be genetically analyzed by using SNP genotyping platforms. After quality control and genetic imputation of these data, polygenic risk scores will be calculated with advanced methods such as continuous shrinkage (90). This approach will allow us to quantitatively estimate the genetic burden of the mental disorders in our sample. Such a genetic load index will be the basis for genetic analyzes of the impact of polygenic risk scores on different clinical traits and the degree of genetic overlap between the various diagnostic groups in the CDP study. In blood RNA collected in PaxGene tubes, we will specifically assess levels of microRNAs and mRNAs, including histone deacetylase 1 and 2. Subsequently, we will perform univariate and multivariate pathway analyzes to identify disturbed genetic and epigenetic pathways within biotype-stratified subgroups of patients. We will investigate all pathways and epigenetic markers with individual models or tests as part of advanced longitudinal and cross-sectional machine learning methods (49).

### 2.12. Longitudinal assessment

The CDP study is mainly a cross-sectional investigation; however, after a successful initiation phase, we will initiate a longitudinal re-assessment with a six-month follow-up only in patients with first-episode SZ and a regular two-year follow-up period in all patients. Moreover, because the study data are embedded in the Munich Mental Health Biobank (52), we will have access to the longitudinal clinical data from participants’ medical records.

## 3. Results

### 3.1. Establishing a deep phenotyping cohort

**Figure 1:**
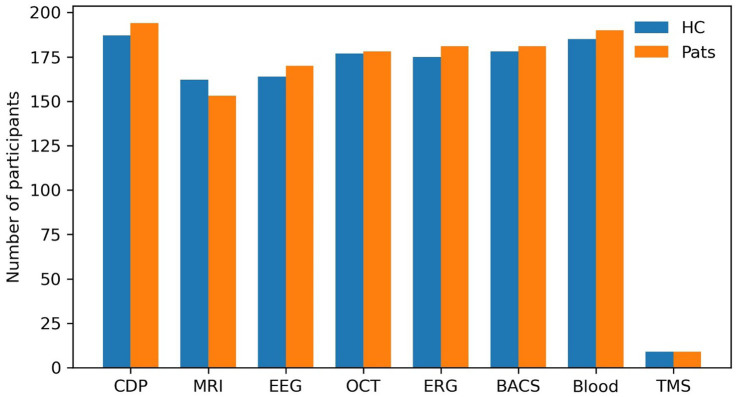
Numbers of patients and healthy controls in the Clinical Deep Phenotyping study grouped by modalities. Bar plots indicate the number of patients (orange bars) and healthy controls (blue bars) in the Clinical Deep Phenotyping (CDP) study who participated in the CDP study in general and in the various study examinations. BACS, Brief Assessment of Cognition in Schizophrenia; CDP, clinical deep phenotyping; EEG, electroencephalography; ERG, electroretinogram; HC, healthy controls; OCT, optical coherence tomography; Pats, patients; MRI, magnetic resonance imaging; TMS, transcranial magnetic stimulation.

From the start of the study on October 1, 2020, until the end of the initiation phase on October 31, 2022, 381 participants were enrolled in the ongoing CDP study. Background characteristics are shown in Table 2, including the numbers of patients for each diagnosis and the numbers of HC and unaffected relatives (UR). Among the patients, 65.5% were male, and among the HC, 46.5%. Table 2 also shows the modalities performed in patients and HC. We performed Fischer’s exact test to investigate whether any CDP assessments were affected by group and found that insufficient evidence is available to show whether the decision to participate or inclusion in any of the mentioned examinations was significantly dependent on whether the participant was a patient or HC (*p* values: MRI, 0.06; EEG, 1.00; OCT, 0.31; ERG, 1.0; BACS, 0.51; blood sampling 0.69; and TMS, 1; Figure 1).

**Table 2:** Participants in the Clinical Deep Phenotyping study.

CDP cohort		
	Healthy controls	Patients
Participants, *n*	187	194
Age, mean (SD), y	34.5 (12.3)	39.5 (11.1)
Female, *n*	100	67
Male, *n*	87	127
DSM-5-TR diagnosis, *n*		
Schizophrenia		110
Schizoaffective disorder		44
Major depression		18
Brief psychotic disorder		6
Drug induced psychosis		5
Delusional disorder		2
Bipolar disorder		9
Unaffected relatives	6	
Modalities, *n*		
BACS test	178	181
MRI	162	153
Resting-state EEG	164	170
P300 (EEG)	162	167
TMS	9	9
ERG	175	181
OCT	177	178
Blood sampling	185	190
PBMC	161	133
hiPSC	10	14
CSF		18
Agreed to be recontacted	178	169
CDP follow-up of MIMICSS participants		
MIMICSS participants, *n*	10	15
Time between MIMICSS and CDP, mean (SD), *y*	5.9 (0.7)	5.7 (1.0)

BACS, Brief Assessment of Cognition in Schizophrenia; CDP, Clinical Deep Phenotyping; CSF, cerebrospinal fluid; DSM5, Diagnostic and Statistical Manual of Mental Disorders; EEG, electroencephalography; ERG, electroretinogram; hiPSC, human induced pluripotent stem cells; MIMICSS, Multimodal Imaging in Chronic Schizophrenia Study; MRI, magnetic resonance imaging; OCT, optical coherence tomography; p300, EEG with an auditory stimulus; PBMC, peripheral blood mononuclear cells; SD, standard deviation; TMS, transcranial magnetic stimulation.

### 3.2. Enabling a longitudinal, translational cohort based on previous studies

The MIMICSS included 154 individuals (76 participants with a diagnosis of SZ, 56 HC, and 22 UR of patients with SZ). MIMICSS participants underwent multimodal imaging and a cognitive test battery. Of the MIMICSS participants, 15 patients with SZ and 10 HC accepted our invitation to join the CDP study. These individuals were enrolled in the CDP study a mean of 5.7 (± 1.0) years and 5.9 (± 0.7) years after their participation in MIMICSS. We continue to invite MIMICSS participants to the CDP study because their participation might allow us to perform longitudinal examinations in a subgroup at the start of the CDP study.

PBMC were isolated from 35 patients with SZ, 20 HC, and 5 UR who participated in the PsyCourse-based hiPSC cohort study. hiPSCs were generated from 20 patients with SZ, 12 HC, and 3 UR (Supplemental Text; Supplementary Table S4).

**Figure 2:**
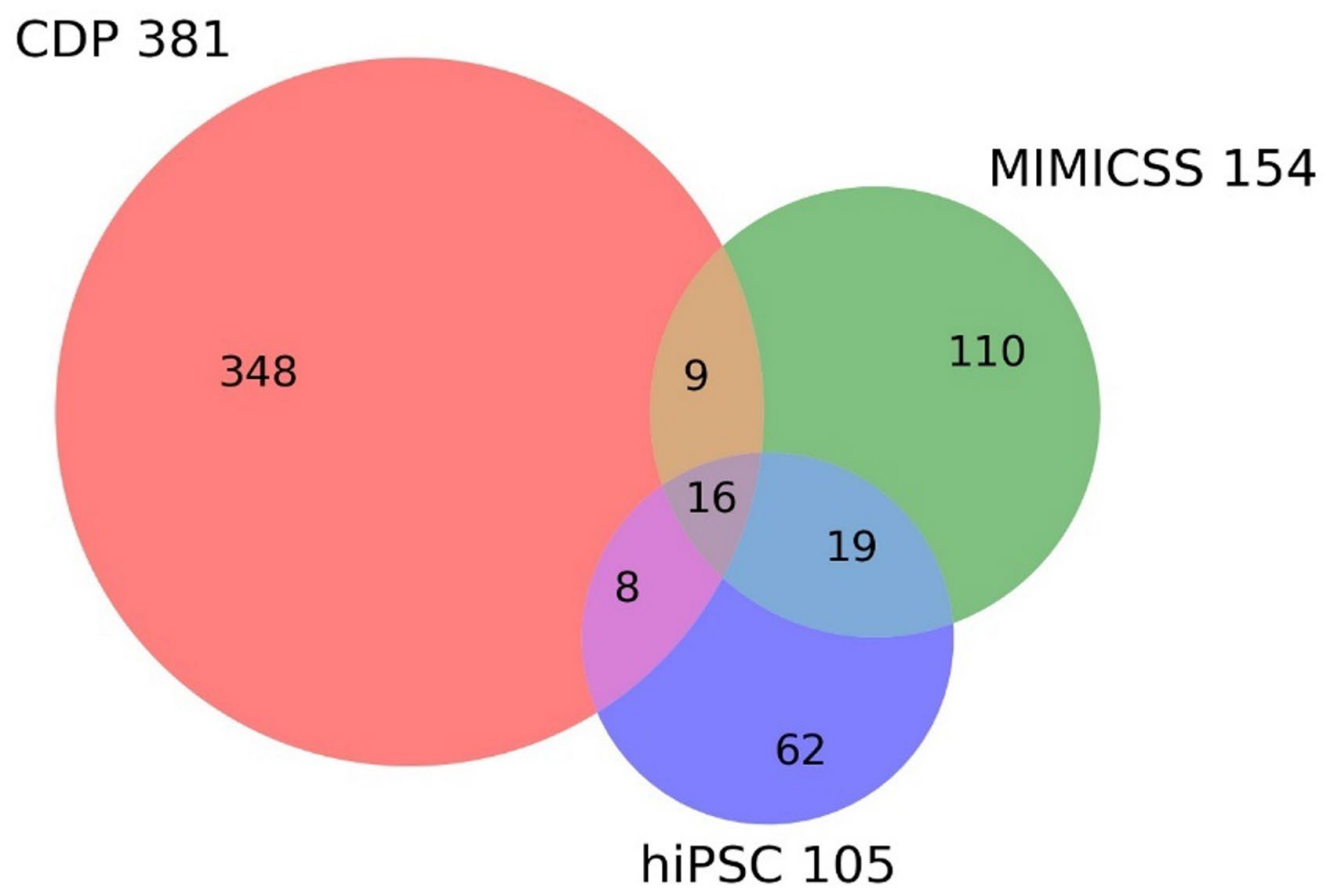
Overlap between longitudinal and translational subcohorts in the Clinical Deep Phenotyping study. The Venn diagram shows the inclusion in the Clinical Deep Phenotyping (CDP) study of participants who previously participated in the Multimodal Imaging in Chronic Schizophrenia Study (MIMICSS) and/or the human induced pluripotent stem cells (hiPSC) cohort of the PsyCourse study. The CDP, MMICSS, and hiPSC cohorts currently have a total of 381, 154, and 105 participants, respectively. CDP, Clinical deep phenotyping; hiPSC, human induced pluripotent stem cells; MIMICSS, Multimodal Imaging in Chronic Schizophrenia Study.

The successful inclusion of MIMICSS participants and participants from the hiPSC cohort from the PsyCourse study enables that the CDP study already contains longitudinal and translational subcohorts (Table 2; Figure 2).

### 3.3. Clinical deep phenotyping study covers several RDoC analysis units

**Figure 3:**
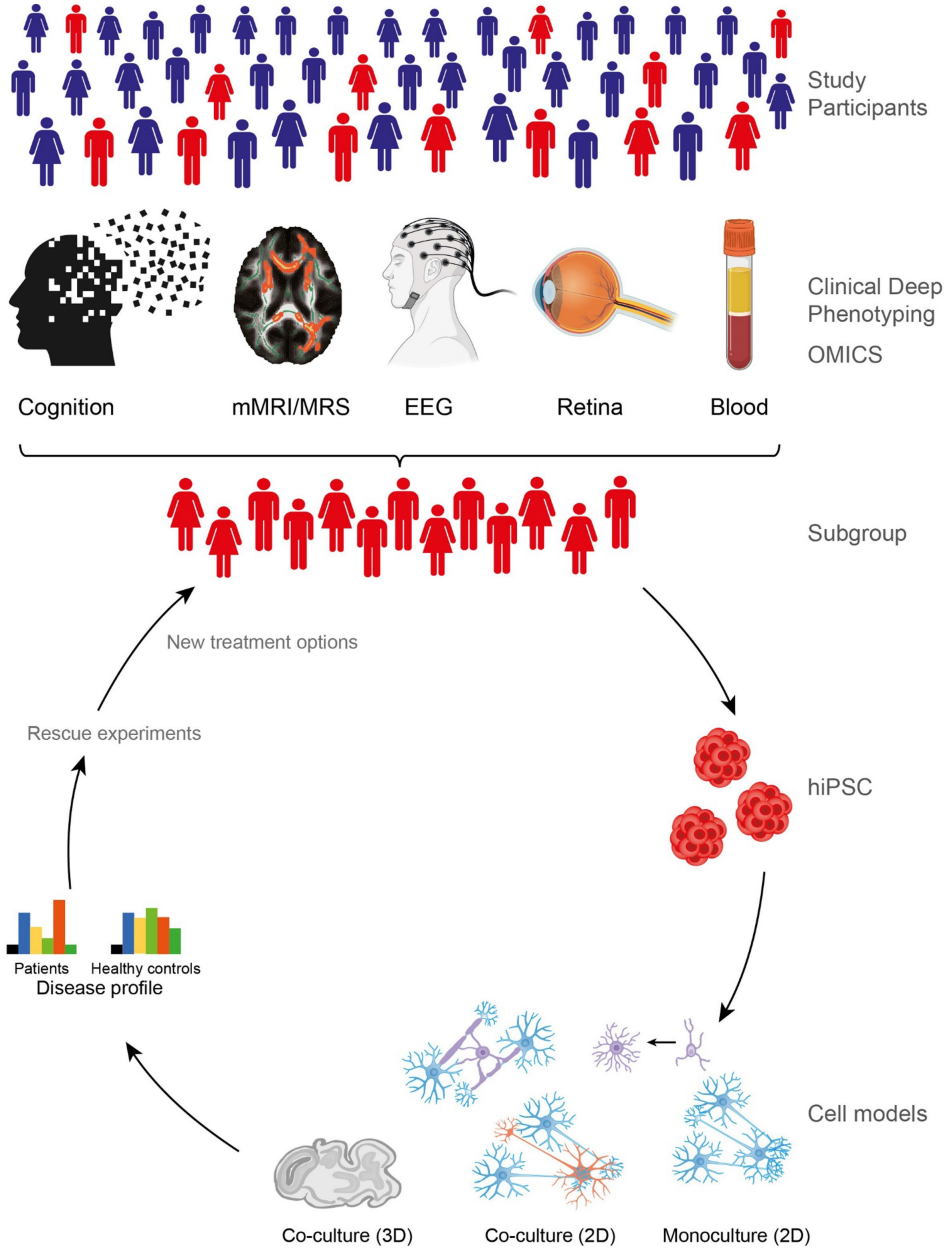
Summary of the approach of the Clinical Deep Phenotyping study. Clinical deep phenotyping of patients with severe mental illness and healthy controls includes cognitive, cerebral, and retinal assessments and blood-based biobanking; when space allows, isolated peripheral blood mononuclear cells are also added to the biobank, enabling later generation of human induced pluripotent stem cells (hiPSCs) from selected participants. After sufficient patient stratification, e.g., based on genetic subtypes and/or subphenotypes, subsequent hiPSC reprogramming from representative patients remains a bottleneck because of the high costs and time required. hiPSC models enable experimental validation and investigations of generated hypotheses in cellular 2D/3D monoculture and co-culture systems to reveal disease-specific molecular profiles. These models also allow treatment options to be screened, paving the way for new treatments that can be introduced into clinical practice after being verified in clinical trials with increasing numbers of patients; such trials are best performed in patient subgroups that are aligned with the initial stratification strategy. Adapted from (84). EEG, electroencephalography; hiPSC, human induced pluripotent stem cells; mMRI, multimodal magnetic resonance imaging; MRS, magnetic resonance spectroscopy.

The multimodal approach of the CDP study is similar to the approach of the RDoC initiative. Thus, all investigations and assessments are performed in all participants independent of their clinical diagnosis. For example, the PANSS is assessed in all patients and HC. In this way, the CDP study, which focuses in particular on the cognitive systems of the RDoC matrix, covers multiple layers of the RDoC analysis units (Genes, Molecules, Cells, Circuits, Physiology, Self-Reports, and paradigms; Table 3) and might provide novel findings on the neurobiological underpinnings of cognitive impairments in SMI (Figure 3).

**Table 3:** Examples of Research Domain Criteria units of analysis in the Clinical Deep Phenotyping study.

RDoC Units of analysis	CDP Source / Method	Underlying principle
Genes	Blood biobanking	Genotyping
Molecules	Blood biobanking	Transcriptomics
		Proteomics
	MRS	Spectroscopy of candidate molecules in specific brain areas
Cells	Blood biobanking	hiPSC-derived brain cells
	OCT imaging	Analysis of retinal cytoarchitecture
Circuits	mMRI	T1, T2, DTI
Physiology	EEG	Auditory stimulus (p300), resting state
	TMS	Short-interval cortical inhibition
	fMRI	Functional resting MRI
Behavior	--	--
Self-reports	PANSS	Positive, negative symptoms
Paradigms	BACS	Cognitive tasks

BACS, Brief Assessment of Cognition in Schizophrenia; CDP, Clinical Deep Phenotyping; DTI, diffusion tensor imaging; EEG, electroencephalography; fMRI, functional resting MRI; hiPSC, human induced pluripotent stem cells; mMRI, multimodal magnetic resonance imaging; MRS, magnetic resonance spectroscopy; OCT, optical coherence tomography; PANSS, Positive and Negative Syndrome Scale; RDoC, Research Domain Criteria; T1, T1-weighted images; T2, T2-weighted images; TMS, transcranial magnetic stimulation.

## 4. Discussion

This article presents the protocol and initiation phase of the ongoing CDP study. Between October 1, 2020, and October 31, 2022, 381 participants, mostly with SSD, were recruited into the CDP cohort. In the CDP study, all participants undergo deep phenotyping, e.g., by multimodal MRI imaging, resting-state EEG, activation EEG, retinal anatomical and electrophysiological measurements, and blood and hiPSC biobanking and postprocessing.

The cross-diagnostic CDP study was inspired by the RDoC initiative (44). Although, the clinical diagnostic systems DSM-5 and ICD-10 do not reflect neurobiology, there are no plans to change them in the near future. Therefore, the CDP study uses both systems in parallel to allow potential clinical translation and aims to reveal the neurobiological underpinnings of clinically relevant subgroups across SMI disease courses, such as treatment resistance, remission, and cognitive impairments, by using a multimodal approach with RDoC-orientated clinical neuroscience tools.

To identify patient subgroups, WHOQOL-BREF and GAF are used to stratify patients according to social functioning and quality of life, both of which can differentiate between genetically different subgroups of psychosis (91). We aim to investigate potential neurobiological alterations in treatment-resistant patients by assessing lifetime clozapine treatment as a proxy (8). Moreover, we categorize remission by applying the established “Andreasen criteria” (51).

Cognitive functioning is often impaired in patients with SSD (92) but is only marginally influenced by antipsychotic treatment (93). Furthermore, it may predict treatment response and remission (94–96). Therefore, one aim of the CDP study is to investigate whether cognitive impairments in SMI are reflected in neurobiological patterns because finding such patterns might help to identify patients at cognitive risk in future investigations or clinical trials.

Previous studies have investigated biological aspects of remission and treatment response. For example, one study found that patients with TRS had a more pronounced reduction in gray matter and lower perfusion of frontotemporal regions than treatment-responsive patients (97). Moreover, another study showed that non-remitted patients with first-episode SZ have smaller hippocampal tail volumes than remitted first-episode patients, whereas hippocampal head and body volumes did not significantly differ between groups (98).

Most studies that investigate SMI from a biological perspective are limited by low sample sizes or the use of only a few assessment modalities. For example, the Enhancing Neuro Imaging Genetics Through Meta-Analysis (ENIGMA) initiative aims to dissect neuropsychiatric disorders by combining only structural MRI, DTI, and fMRI data with genetic analyzes in large-scale cohorts (99). On the other hand, studies with retinal assessments, which represent an easily accessible window to the CNS and provide high-resolution data that might help to provide a deeper pathophysiological understanding, use mostly only methods such as OCT and ERG (84, 100). The only study to date that used both retinal and cerebral assessments in the same individuals had a low sample size (*n* = 24), which limited subsequent subgroup stratification (101). To enable the identification of clinically relevant subgroups, we aim to overcome these disadvantages in study designs (102, 103) and to consider the variability within and across individuals by performing deep phenotyping in over 500 participants with SMI.

To study and validate whether clinically relevant subgroups are reflected, at least to a certain extent, by altered biotypes, we aim to analyze multimodal data from the CDP assessments, including brain and retinal electrophysiology and anatomy and neurocognitive data, and combine them with blood- and CSF-derived data, such as transcriptomics and proteomics, and genetic information. This aim will be supported by biobanking of biomaterial from CDP participants at the Munich Mental Health Biobank.

Brain structure is heritable (104), and twin and family studies in UR show that UR have brain volume abnormalities similar to those found in patients (105). Moreover, SMI are associated with global brain structure alterations (106). For this reason, the CDP uses multiple brain imaging modalities to investigate the underlying anatomy and physiology.

The cross-diagnostic design of the CDP study will allow us to not only evaluate differences in the results of each type of assessment between patients with SMI and HC and between subgroups of patients with SMI, but also to examine the complex relations between the assessed modalities. We understand multilevel research as the simultaneous investigation of different domains of neurophysiological investigations and the subsequent confirmation of plausible findings, e.g., the significant distinction between patients and HC. Content validity is increased if matches are shown, e.g., in regions of the frontal brain, and reflected across modalities (e.g., structural alterations in mMRI and electrophysiological alterations in EEG). Furthermore, MRS can be used to distinguish between regional excitatory and inhibitory effects.

Environmental factors also play an important role in structural brain alterations (107). One confounding factor that may influence brain volume is medication intake, and it is difficult to determine whether brain volume changes are a consequence of disease-specific processes or antipsychotic treatment (108). Taking into account the confounding role of psychotropic drugs, the CDP study records current and past drug intake in all participants. To disentangle the complex nature of morphological and functional brain changes in SMI and control for antipsychotic treatment effects that might impact physiological parameters or blood–brain barrier alterations, for example, we intend to include also a substantial number of drug-naïve and first-episode patients in the CDP cohort.

The German national schizophrenia guidelines recommend that a lumbar puncture with routine CSF analysis is performed in all patients with the first episode of an SMI.^3^ Of interest in this context is a large-scale retrospective study that postulated that CSF shows distinct, psychosis-specific patterns that include markers of inflammation or infection (109, 110). Hence, when clinically indicated, lumbar punctures are performed in a substantial subgroup of CDP patients to investigate CSF signatures in patients with SMI and assess the associations of such signatures with other assessed modalities (i.e., imaging, electrophysiology, and cognitive performance). To date, no large-scale cross-sectional study has examined the relationship between cognitive performance and CSF abnormalities in SMI. Furthermore, we aim to conduct an RDoC-conform longitudinal observational follow-up in patients with SMI to assess neuroinflammatory markers and glia-derived neurotrophic factors in CSF and the effect of these substances on cognition and symptomatic outcomes over the course of the disease. Moreover, in a subgroup of patients with SSD we also aim to evaluate the blood–brain barrier *via* contrast-enhanced MRI.

### 4.1. Relationship of the CDP study to international cohort studies

Comparability of the CDP study with other large cohort studies, such as NAKO (German National Cohort Study), ENIGMA (The Enhancing NeuroImaging Genetics through Meta-Analysis), the United Kingdom Biobank (United Kingdom Biobank), and the HCP, offers the possibility to study the relationship between the CDP data and those of much larger samples (99, 111–113). For example, ENIGMA provides data on various disorders, including SZ and MDD (114). Previously published work shows multicenter efforts to link genetics to brain structures, for example (106). Most recently, a multicenter ENIGMA effort identified 15 “hotspots” in the genome that either accelerate or slow brain aging–a finding that could potentially provide new targets for medications for psychiatric disorders (106). The CDP study uses a 3 T Prisma Magnetom Siemens scanner and the same MRI protocols as used in the HCP sample (75) and thus provides technically good conditions for obtaining normative reference values for multimodal MRI recordings. The HCP and CDP study collect similar cognition parameters and sociodemographic information. Thus, the use of the HCP protocol for multimodal MRI also allows direct comparison of the CDP sample with the HCP lifespan samples, the HCP young adult S1200 sample, and the HCP aging sample, covering individuals aged from 5 to over 100 years (75, 115).^4^ In the future, clinical HCP studies will also allow for direct comparison and referencing of clinical diseases.

### 4.2. Validating the potential of retina measurements as a window to the brain

Numerous studies on neurodegenerative disorders, including multiple sclerosis (116), Alzheimer disease (117), and Parkinson disease (118), have applied retinal OCT to assess how the retinal nerve fiber layer gradually thins. Interestingly, the retina shows typical changes in neurochemistry, morphology, electrophysiology, and function that reflect several pathomechanisms of neurodegenerative disorders and stroke (81).

Although OCT and ERG are broadly available, quick evaluation techniques, retinal measurements are not an established feature of research in biological psychiatry and are a long way from being used as diagnostic tools. Nevertheless, recent meta-analyzes provided evidence for the phenomenon of retinal thinning in SZ and BD (82–85). Moreover, one study found that the outer nuclear layer, which was altered in psychosis, was associated with total brain and white matter volume in a small cohort of 25 patients with psychosis and 15 HC (101).

Of note, the majority of retinal studies in SMI are limited by small sample sizes. Therefore, the large-scale CDP study aims to deliver further evidence for the potential and feasibility of retinal investigations in psychiatric research by validating the initial findings of retinal alterations presented here in the full CDP cohort.

As a preliminary finding from the CDP study, we recently published OCT findings in 65 patients with an SSD and 72 HC that provided evidence of thinner inner retinal layers and thinner total macular thickness in SSDs (119). These changes could not be explained by comorbidities such as hypertension, diabetes, or higher body mass index (BMI), all of which also affect retinal thickness and are enriched in patients with SSD.

As the next step, the CDP study will investigate in the future to what extent the retinal findings are related to brain-based CDP modalities and whether retinal investigations could be used as follow-up investigations.

### 4.3. Bridging the translational gap of micro- and macrocircuit research

Previously, the biological causes of SMI could be studied only by examining peripheral tissues, comparing imaging results with other findings, comparing genetic data, and analyzing postmortem brain samples. However, there is now great optimism that hiPSCs (120) will allow researchers to create almost any type of neuronal or glial cell and thus perform *in vitro* research on the brain. Technologies related to hiPSCs are expected to lead to advances in translational psychiatry (89, 121). To date, hiPSC models have enabled the investigation of hypotheses from GWASs, which have found more than 200 genes with a potential role in SZ (122, 123). Studies of hiPSCs have shown dysfunctions in neurons and glial cells in SZ (88, 121, 124). Currently, most hiPSC experiments enable identification of only basic clinical features, in particular variables such as age and diagnosis. Reports of genetic findings are rare and do not describe detailed clinical features. Thus, patient samples with extensive data on a broad range of characteristics are required to enable translation of clinical findings to the laboratory and from the laboratory back into clinical practice (89). Such extensively characterized samples would enable us to understand the underlying biology of neuropsychiatric diseases such as SZ and translate the biological findings into clinically relevant phenotypes. Therefore, we aim to use representative subgroups of our deeply phenotyped cohort to close the translational gap between hiPSC models and clinical symptomatology in patients. To this end, we will apply stratification strategies with deep learning algorithms based on the examined multi-layer data. Thus, after performing big data analysis, we will evaluate only meaningful subgroups of representative patients with hiPSC-based technology (89). In the long term, by using initial stratification strategies we expect to be able to develop new personalized therapeutic approaches with the help of clusters that are built from examined datasets with an RDoC approach and also with the help of patient-derived cell systems. We believe that this approach will help to push the boundaries of translational psychiatry (89, 125).

### 4.4. Summary and outlook

In summary, the multi-and interdisciplinary CDP study aims to non-invasively map the CNS in detail at different levels by using various examinations of the brain and retina to gain biological insights into disease patterns and manifestations in SMI and to merge them with genetic, cellular, clinical, and cognitive data. The study follows a confirmatory approach that aims on the one hand to find multimodal similarities and differences in terms of content and, on the other hand, to examine how our study data relate to those of larger cohorts. In small, well-designed subsamples, we aim to integrate our macroscopic assessments with hiPSC-based *in vitro* investigations and examinations of inflammatory markers in blood, brain, and CSF.

As mentioned above, so far only preliminary retinal data from the CDP cohort have been published (119) because the sample size is not large enough to obtain sound results for all the variables examined. Of note, similar to the data in the initial OCT paper (119), we plan to make published data available to enable open research exchange. However, the fact that no further preliminary findings have been published is a limitation of the current status of the CDP study. In the long term, we plan to pool our data with data from other centers and to participate in global efforts to better understand brain structure and function and cellular mechanisms in SMI by using multivariate data. The CDP study might support the scientific endeavor to identify neurobiology-informed SMI subgroups of patients who could benefit from personalized and tailored treatment in the future.

## Data availability statement

The original contributions presented in the study are included in the article/Supplementary material, further inquiries can be directed to the corresponding author.

## Ethics statement

The studies involving human participants were reviewed and approved by the Ethics Committee of the Medical Faculty of the Ludwig-Maximilian-University, number: 20–528. The patients/participants provided their written informed consent to participate in this study.

## Author contributions

DK, EW, AS, BM, and FR designed and conceptualized the CDP study. LK, EB, KH, IJ, GI, JMe, JMo, and VG recruited patients and collected study data. EW trained staff on diagnostic and clinical assessments. LK performed data preparation and statistical analysis. LK and FR performed data visualization. LK, DK, AS, PF, and FR wrote the first draft of the manuscript. All authors contributed to and approved the final manuscript.

## Funding

This research was supported by BMBF with the EraNet project GDNF UpReg (01EW2206) to PF. PF, AS, LK, and FR were supported by the Else Kröner-Fresenius Foundation Research College “Translational Psychiatry” and the Residency/PhD track of the International Max Planck Research School for Translational Psychiatry (IMPRS-TP). FR is supported by the Munich Clinician Scientist Program (MCSP) of the Faculty of Medicine at LMU Munich (FöFoLePlus Reg.-Nr. 09/2019), Braun-Stiftung (BBST-D-20-00032) and the Lisa Oehler-Stiftung (2022–2023). MC is supported by the Support Program for Research and Teaching (FöFoLe) research project (registration no. 1130). JS was endorsed by the BMBF funded project DZPG (German Center of Mental Health), partner site Halle-Jena-Magdeburg (01EE2305D, 01EE2103, co-funded by Saxony-Anhalt’s Ministry of Economy, Science and Digitalisation: I 212), Germany, and PF was endorsed by the DZPG (German Center of Mental Health), partner site Munich-Augsburg (01EE2303F and 01EE2303A).

## Conflict of interest

The authors declare that the research was conducted in the absence of any commercial or financial relationships that could be construed as a potential conflict of interest.

## Publisher’s note

All claims expressed in this article are solely those of the authors and do not necessarily represent those of their affiliated organizations, or those of the publisher, the editors and the reviewers. Any product that may be evaluated in this article, or claim that may be made by its manufacturer, is not guaranteed or endorsed by the publisher.

## Footnotes

^1^National Institute of Mental Health. Definitions of the RDoC Domains and Constructs. https://www.nimh.nih.gov/research/research-funded-by-nimh/rdoc/definitions-of-the-rdoc-domains-and-constructs (Accessed January 01, 2023).

^2^National Institute of Mental Health. RDoC Matrix. Important Notes on the Matrix. https://www.nimh.nih.gov/research/research-funded-by-nimh/rdoc/constructs/rdoc-matrix (Accessed January 01, 2023).

^3^AWMF online. S3-Leitlinie Schizophrenie. https://register.awmf.org/de/leitlinien/detail/038-009 (Accessed January 01, 2023).

^4^Connectome coordination facility. 1,200 Subjects Data Release. https://www.humanconnectome.org/study/hcp-young-adult/document/1200-subjects-data-release (Accessed January 01, 2023).

## Abbreviations

ACC: anterior cingulate cortex
BACS: Brief Assessment of Cognition in Schizophrenia
BD: bipolar disorder
BrPsyD: brief psychotic disorder
CDP: Clinical Deep Phenotyping
CDSS: Calgary Depression Rating Scale for Schizophrenia
CGI: Clinical Global Impression
CSF: cerebrospinal fluid
CTQ-Screen: Childhood Trauma Screener
DD: delusional disorder
DIP: drug-induced psychosis
DLPFC: left dorsolateral prefrontal cortex
DSM-5: Diagnostic and Statistical Manual of Mental Disorders, 5th Edition
DTI: diffusion tensor imaging
EEG: electroencephalography
EMG: surface electromyography
ENIGMA: Enhancing Neuro Imaging Genetics Through Meta-Analysis
ERG: electroretinography
FRPS: Framingham Risk Prediction Score
GAF: Global Assessment of Functioning
GWASs: genome-wide association studies
HC: healthy controls
HCP: Human Connectome Project
ICD-10: International Classification of Diseases, 10th revision
IDS-C30: Inventory of Depressive Symptomatology version with 30 items
M. I. N. I.: Mini-International Neuropsychiatric Interview
MCTQ: Munich Chronotype Questionnaire
MDD: major depressive disorder
MEPs: motor evoked potentials
MIMICSS: Multimodal Imaging in Chronic Schizophrenia Study
MRS: magnetic resonance spectroscopy
NAKO: German National Cohort Study
OCT: optical coherence tomography
OCT-A: OCT angiography
PANSS: Positive and Negative Syndrome Scale
PBMC: peripheral blood mononuclear cells
PHQ-9: Patient Health Questionnaire – 9
PROCAM: Prospective Cardiovascular Münster Score
RDoC: Research Domain Criteria
RSWG: Remission in Schizophrenia Working Group
SMI: severe mental illness
SNP: single nucleotide polymorphism
SSD: schizophrenia spectrum disorder
SZ: schizophrenia
SZA: schizoaffective disorder
T1-MPRAGE: T1-weighted magnetization prepared-rapid acquisition gradient echo
T2-FLAIR: T2-weighted-fluid-attenuated inversion recovery
T2-SPACE: T2 sampling perfection with application-optimized contrasts using different flip angle evolution
TMS: transcranial magnetic stimulation
TRS: treatment-resistant schizophrenia
UR: unaffected relatives
WHO-5: World Health Organization-5 Well-Being Index
WHOQOL-BREF: World Health Organization Quality of Life Scale, abbreviated version
YMRS: Young Mania Rating Scale
fMRI: task-based functional MRI
hiPSCs: human induced pluripotent stem cells
mMRI: multimodal magnetic resonance imaging
r_g_: genetic correlation
rsfMRI: resting-state functional MRI
