# Supplementary material for: The multimodal Munich Clinical Deep Phenotyping study to bridge the translational gap in severe mental illness treatment research
Source: Front Psychiatry. 2023 May 5;14:1179811. doi: 10.3389/fpsyt.2023.1179811 (PMC10196006; doi:10.3389/fpsyt.2023.1179811)
Supplement: Supplementary file 1 [file Data_Sheet_1.PDF]

Supplementary Material

1 Supplementary Text

The Multimodal Imaging in Chronic Schizophrenia Study (MIMICSS) was a pilot study based on the longitudinal PsyCourse study (Budde et al., 2019; local ethics committee, LMU Munich, Munich, Germany project number: 17-13). The MIMICSS focused on cognitive deficits in patients with schizophrenia (SZ) and was performed from September 16, 2014, to April 3, 2018, at the Department of Psychiatry and Psychotherapy, LMU Munich, Germany.

The MIMICSS included 56 healthy controls (HC; 40 men and 16 women), 76 patients with SZ; 59 men and 17 women and 22 unaffected relatives (UR; 6 men and 16 women) of patients with SZ. The mean age of the patients with SZ was 34.8 years (± 11.7 years); of the HC, 33.3 years (± 12.1 years); and of the UR, 42.7 years (± 17.0 years).

Clinical phenotyping included assessment of the medical history; a structured interview to assess psychopathology that included the Positive and Negative Syndrome Scale (PANSS), Clinical Global Impression (CGI), Global Assessment of Functioning (GAF), Beck Depression Inventory Second Edition (BDI-II), Scale for the Assessment of Negative Symptoms (SANS), Social Adjustment Scale II (SAS-II)), neurocognitive testing (Verbal Learning Memory Test (VLMT), Trail Making Test, part A (TMT-A), Trail Making Test, part B (TMT-B), Digit-Span-Task, Digit Symbol Substitution Test (DSST), and Test of Attention (d2 Test); and multimodal MRI imaging (mMRI). Moreover, peripheral blood mononuclear cells (PBMC) were isolated from individuals (35 patients with SZ, 20 HC, and 5 UR) who also participated in the PsyCourse-based human induced pluripotent stem cells (hiPSCs) cohort study (ethics committee project number: 17-880), allowing hiPSCs to be generated from 20 patients with SZ, 12 HC, and 3 UR (Table S4).

2 Supplementary Figures and Tables

2.1 Supplementary Tables

Table S1. Overview of assessment modalities in the Clinical Deep Phenotyping study

**Basic Assessment**
**Clinical characterization**
Basic phenotyping: Munich Mental Health Biobank
Socioeconomic assessment*
History of present illness
Current and past medications
Substance use history
**Transdiagnostic self-ratings**
Childhood Trauma Screener (CTQ-Screen)
Brief Resilience Scale
Loneliness Scale
Lubben Social Network Scale
WHO Well-Being Index scale (WHO-5)
Patient Health Questionnaire-9 (PHQ-9)
Munich Chronotype Questionnaire (MCTQ)
WHO Quality of Life Scale (WHOQOL-BREF)
Medical History
Inpatient/outpatient status
If current outpatient: time of last hospitalization because of the illness
Mini International Neuropsychiatric Interview (M.I.N.I. interview, DSM-5-TR)
Inclusion/exclusion criteria**
**Psychiatric history**
Age of onset
Duration of untreated illness
Duration of illness
First contact with psychiatrist because of the illness
Number of hospitalizations because of the illness
Number and duration of illness episodes
Currently in first episode (yes/no)
Current clozapine user, duration of clozapine treatment, past clozapine use
Psychiatric medication (current, lifetime)
**Physical assessment**
Medication (current)
Structured assessment of past and current physical comorbidities***
Prospective Cardiovascular Münster Study (PROCAM) score
Framingham-Risk Prediction Score (FRPS)
Ophthalmological screening to assess potential influence on OCT/ERG parameters

Shift work/Crossed a time zone with > 2 h difference within last month, yes/no				
Disease-related scales				
Positive and Negative Syndrome Scale (PANSS)				
PANSS RSWG criteria (“Andreasen criteria”)				
Calgary Depression Rating Scale for Schizophrenia (CDSS)				
Young Mania Rating Scale (YMRS)				
Inventory of Depressive Symptomatology clinician-rated version with 30 items (IDS-C30)				
Global Assessment of Functioning (GAF)				
Clinical Global Impression (CGI)				
Edinburgh Handedness Inventory, short form				
Fagerström test (for all smokers) self-rating scale				
Cognitive assessment				
Brief Assessment of Cognition in Schizophrenia (BACS)				
Cerebral Assessment				
Multimodal magnetic resonance imaging (mMRI) ****				
Electroencephalography (EEG)				
Transcranial magnetic stimulation (TMS)				
Retinal assessment				
Optical coherence tomography (OCT)/OCT angiography (OCT-A)				
Electroretinogram (ERG)				
Biobanking (Munich Mental Health Biobank)				
**Overview of Biosamples**	**Supplier**	**CatNo**	**Purpose**	**Storage temperature**
Pax Gene blood RNA tube	BD	762165	RNA	-80°C
7.5-mL K3EDTA Monovette	Sarstedt	01.1605.001	DNA	-80°C
9-mL K3EDTA Monovette	Sarstedt	02.1066.001	Plasma	-80°C
9-mL Serum S-Monovette	Sarstedt	02.1063.001	Serum	-80°C
BD Vacutainer 10-mL Glass Sodium Heparin Tubes	BD	368480	PBMC	-176°C

Abbreviations: CDP, clinical deep phenotyping; DSM-5-TR, Diagnostic and Statistical Manual of Mental Disorders, 5th Edition, text revision; OCT, optical coherence tomography; ERG, electroretinogram; RSWG, Remission in Schizophrenia Working Group; EDTA; ethylene diamine tetraacetic acid; CatNo; catalogue number; DNA, deoxyribonucleic acid; RNA, ribonucleic acid; PBMC; peripheral blood mononuclear cells

*Structured sociodemographic assessment (e.g., age, sex, job situation, relationship status, highest educational degree, number of school years, number of years of professional training, current living situation, ethnic origin)

**Based on the M.I.N.I., and for neurodegenerative diseases, based on patient files/clinical impression

*** Structured assessment of past and current physical comorbidities, e.g., brain injuries/trauma; chronic diseases, e.g., type I or II diabetes; eye conditions, e.g. glaucoma; arterial hypertension, high-density lipoprotein (HDL)/low-density lipoprotein (LDL) cholesterol and triglyceride abnormalities, if known; body mass index (BMI)

**** mMRI: anatomical MRI measurements, i.e., T1-weighted magnetization prepared-rapid acquisition gradient echo (T1-MPRAGE), T2 sampling perfection with application-optimized contrasts using different flip angle evolution (T2-SPACE), T2-weighted-fluid-attenuated inversion recovery (T2-FLAIR), and diffusion tensor imaging (DTI) and functional MRI measurements, i.e., resting-state functional MRI (rsfMRI), task-based functional MRI (fMRI), and magnetic resonance spectroscopy (MRS)

Table S2. Multimodal magnetic resonance imaging protocol

	**T1-weighted**	**T2-weighted**	**MRS spectroscopy**	**EPI (resting-state AP & PA)**	**EPI (task-fMRI PA)**	**Field maps (AP & PA)**	**Diffusion weighted**
**Sequence**	MP-RAGE	SPACE	CMRR Spectroscopy[2]	CMRR Multiband EPI[1]	CMRR Multiband EPI[1]	SpinEchoField Map	Diff 95dir
**TR, ms**	2500	3200	3000	800	800	8000	5500
**TE, ms**	2.2	564	68	37	37	66	99.2
**Flip angle**	8	120	120-130	52	52	90	90
**B value (s/mm2)**	-	-	-	-	-	-	0/2000
**Diffusion directions**	-	-	-	-	-	-	95
**Resolution, mm^3**	0.8 x 0.8 x 0.8	0.8 x 0.8 x 0.8	30x30x15 (DLPFC), 20x20x20 (ACC)	2 x 2 x 2	2 x 2 x 2	2 x 2 x 2	1.6 x 1.6 x 1.6
**Thickness**	0.8	0.8	-	2	2	2	2
**Accelerator/ multiband factor**	2/-	2/-	-/-	-/8	-/8	-/1	-/3
**PAT mode**	GRAPPA	GRAPPA	-				
**Field of view**	256	256	-	208	208	208	224
**Number of slices**	208	208	-	208	208	72	90
**Imaging direction**	Sagittal	Sagittal	-	Axial	Axial	Axial	Axial
**Duration, min**	06:54	05:57	0:12/1:33<br>6:51/1:09	2x 6:40	10:18	4x 1:00	9:10/0:28

**Measurements**	1	1	143	2x 488	760	2x AP<br>2x PA	2
**Total time, min**	59:52:00						

Abbreviations: ACC, anterior cingulate cortex; AP, anterior-posterior direction; B value, factor that reflects the strength and timing of the gradients used to generate diffusion-weighted images; CMRR, Center for Magnetic Resonance Research, Department of Radiology, University of Minnesota; Diff 95dir, 95-direction diffusion; DLPFC, dorsolateral prefrontal cortex; EPI, echo planar image; fMRI, functional magnetic resonance imaging; GRAPPA, GeneRalized Autocalibrating Partial Parallel Acquisition; MP-RAGE, magnetization prepared-rapid acquisition gradient echo; MRS, magnetic resonance spectroscopy; PA, posterior-anterior direction; PAT, Parallel Acquisition Techniques; SPACE, sampling perfection with application-optimized contrasts using different flip angle evolution; SpinEchoField Map, two simple T2*-weighted images using the gradient echo method; T1, T1-weighted images; T2, T2-weighted images; TE, echo time; TR, repetition time

Applied HCP sequences: https://www.humanconnectome.org/study/hcp-lifespan-aging/project-protocol/imaging-protocols-hcp-aging,[1]https://www.cmrr.umn.edu/multiband/, https://www.cmrr.umn.edu/spectro/

Table S3: Transcranial magnetic stimulation protocol

**Investigation**	**Protocols**	**Time**
TMS-EMG	Application of electrodes; search for the motor cortex “hot-spot” to be stimulated	15:00
	TMS-EMG (left primary motor cortex): MEP, RMT, S1mV, SICI, LICI, ICF.	45:00
	pT-TMS estimation	05:00
		**60:00 (total)**

Abbreviations: ICF, intracortical facilitation; LICI, long interval intracortical inhibition; MEP, motor evoked potentials; pT-TMS, pico-Tesla TMS; RMT, resting motor threshold; S1mV, intensity to evoke a 1mV MEP; SICI, short-interval intracortical inhibition; TMS-EMG, transcranial magnetic stimulation-electromyography

Table S4: Modalities examined in the Multimodal Imaging in Chronic Schizophrenia Study

**MIMICSS Cohort**			
	**HC**	**SZ**	**UR**
Participants	56	76	22
Age, mean (SD), y	33.3 (12.1)	34.8 (11.7)	42.7 (17.0)
Female	16	17	16
Male	40	59	6
**Multimodal MRI imaging, N**			
DTI	55	68	20
rsfMRI1	56	71	20
rsfMRI2	55	70	20
T1	56	71	20
T2	56	71	20
**Blood sampling, PBMC, hiPSC**			
Isolated PBMC	20	35	5
Generated hiPSC	12	20	3
**Follow up of MIMICSS cohort**			
Follow-up period in MIMICSS, mean (SD), y	1.7 (0.5)	2.1 (0.5)	2.2
Age, mean (SD), y	33.7 (10.8)	39.8 (10.8)	53.2 (0.6)
**Multimodal MRI imaging, follow-up**			
DTI	7	17	2
MRS	7	18	2
rsfMRI1	27	18	2
rsfMRI2	27	18	2
T1	27	18	2

Abbreviations: DTI, diffusion tensor imaging; HC, healthy controls; hiPSC, human induced pluripotent stem cells; MIMICSS, Multimodal Imaging in Chronic Schizophrenia Study; MRI, magnetic resonance imaging; MRS, magnetic resonance spectroscopy; PBMC, peripheral blood mononuclear cell; rsfMRI, resting-state functional magnetic resonance imaging; SZ, schizophrenia disorder; T1, T1-weighted images; T2, T2-weighted images; UR, unaffected relatives

2.2 Supplementary Figures

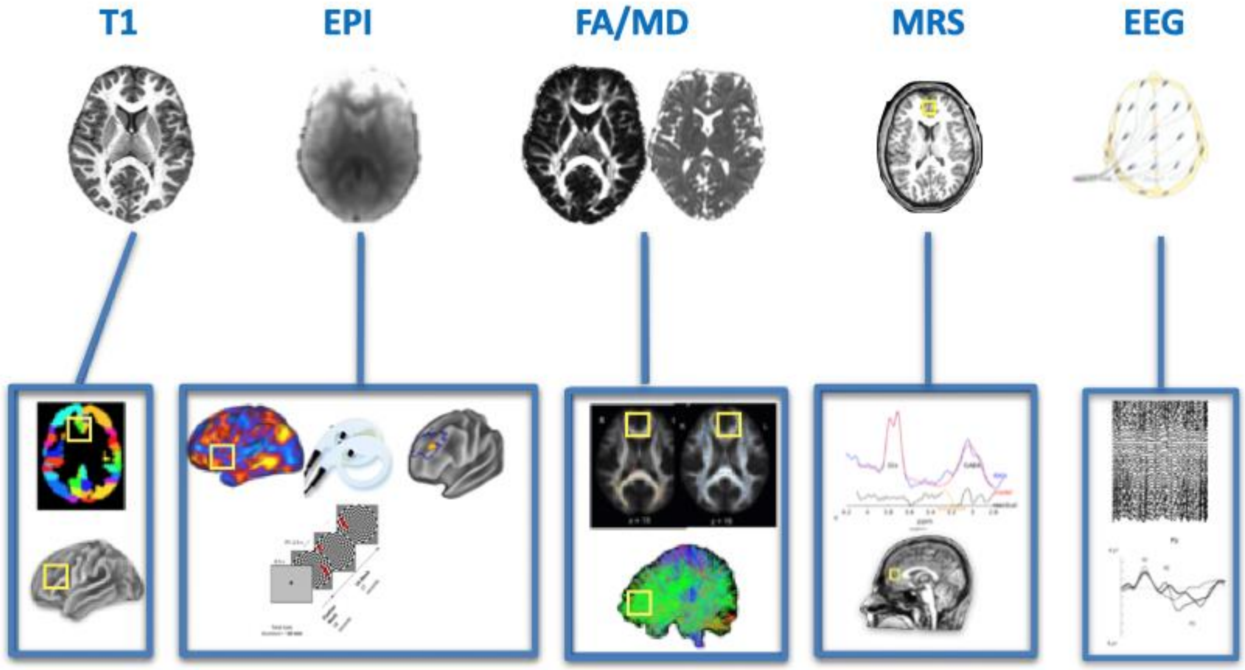

Figure S1. Multimodal magnetic resonance imaging with the subgenual anterior cingulate cortex / dorsal anterior cingulate cortex as regions of interest across modalities

A T-2 weighted sequence is also examined; the task includes a visuomotor task adapted from the Human Connectome Project.

Abbreviations: EEG, electroencephalography; EPI, echo planar imaging (task-functional magnetic resonance imaging [MRI]), FA, fractional anisotropy (diffusion tensor imaging [DTI]); MD, mean diffusivity (DTI); MRS, magnetic resonance spectroscopy (glutamate, gamma aminobutyric acid); T1, magnetization-prepared rapid acquisition with gradient echo (MPRAGE)
